# Two progressed malignant phyllodes tumors of the breast harbor alterations in genes frequently involved in other advanced cancers

**DOI:** 10.1186/s13023-021-01986-z

**Published:** 2021-08-16

**Authors:** Mattea Reinisch, Sherko Kuemmel, Elisabeth Breit, Ingo Theuerkauf, Hakima Harrach, Dorothea Schindowski, Detlef Moka, Marcus Bettstetter, Simona Bruzas, Ouafaa Chiari

**Affiliations:** 1 Breast Unit, Kliniken Essen-Mitte, 45136 Essen, Germany; 2 Department of Gynecology with Breast Center, Charité – Universitätsmedizin Berlin, Corporate Member of Freie Universität Berlin and Humboldt- Universität zu Berlin, 10117 Berlin, Germany; 3 Institute for Pathology Viersen, 41747 Viersen, Germany; 4 Nuclear Medicine Centre, 45136 Essen, Germany; 5 Teilgemeinschaftspraxis Molekularpathologie Südbayern, 81539 München, Germany

**Keywords:** Malignant phyllodes tumor, Breast, Next-generation sequencing, Advanced cancer, Metastasis, Molecular profiling

## Abstract

**Background:**

The genomic landscape of phyllodes tumors (PTs) of the breast is not well defined, especially in patients with advanced disease. To shed light on this topic, paired primary and progressed tumor samples from two patients with malignant PTs were subjected to next-generation sequencing (NGS) followed by functional analysis of genetic alterations using two prediction tools.

**Methods:**

The DNA of both the primary tumor and distant metastases of Patient 1 and the primary and recurrent tumor of Patient 2 were subjected to molecular profiling. NGS with the FoundationOne® assay was performed in a commercial molecular pathology laboratory. Two in silico prediction tools were used to estimate the pathogenicity of indicated genetic alterations.

**Results:**

In total, 38 genomic alterations were detected, of which 11 were predicted to be probably benign. In Patient 1, 14 aberrations were identified in the primary tumor and 17 in pulmonary metastases, 12 of which were identical. In the primary and recurrent tumor of Patient 2, 17 and 15 sequence variants, respectively, were found, with 13 overlapping findings. Affected genes included seven (*TP53*, *TERT*, *APC*, *ARID1A*, *EGFR*, *KMT2D*, and *RB1*) of the top 10 most frequently altered genes in other advanced cancer entities, as well as four actionable therapeutic targets (*EGFR*, *KIT*, *PDGFRA*, and *BRIP1*). Of note, seven genes coding for receptor tyrosine kinases were affected: three in Patient 1 and four in Patient 2. Several genes (e.g. *EPHA3*, *EPHA7*, and *EPHB1*) were shown to be altered for the first time in PTs.

**Conclusions:**

The two progressed malignant PTs investigated here share some of the major genetic events occurring in other advanced cancers.

**Supplementary Information:**

The online version contains supplementary material available at 10.1186/s13023-021-01986-z.

## Background

Phyllodes tumors (PTs) of the breast are rare fibroepithelial neoplasms composed of connective tissue stroma and epithelial elements [1, 2]. The mean age at diagnosis is around 40 years [1]. PTs represent up to 0.5% of all breast tumors and are categorized into benign, borderline, and malignant subtypes. Malignant PTs (MPTs) account for 10–15% of all PT cases [3], and patients with MTPs have a 5‐year survival rate of about 54% [4]. Some patients present with a rapidly growing MTP [5]. Recurrent disease occurs in 23–30% [1] and distant metastases in 9–27%, mainly in MPTs [6]. Patients with metastatic MPTs have a very poor prognosis and may not respond well to standard systemic therapy; the duration of survival after diagnosis of distant disease ranged from 1 to 41 months [6]. The mainstay of treatment for MPTs is local excision of the tumor, aiming to achieve wide negative margins. Systemic adjuvant therapy is not generally recommended for PTs, mainly due to a lack of supporting clinical studies [7]; however, metastatic PTs are treated like soft tissue sarcomas [1, 8] and should therefore receive one or more lines of chemotherapy.

The pathogenesis and underlying genomic landscape of PTs are poorly understood, especially for metastatic disease [9, 10]. The application of commercially available, advanced DNA sequencing technologies has enabled standardized investigation of the mutational status of several hundred cancer-related genes in PTs during the last 8 years and has provided new information on this topic [9–18]. In addition to genetic alterations in classic tumor suppressor genes (TSGs; e.g. *TP53*, *RB1*) or oncogenes (e.g. *EGFR*), hotspot mutations in *PIK3CA* [12], the *TERT* promoter region [18, 19], and *MED12* [9, 14] have frequently been reported. Genetic alterations including loss-of-function alterations in *TP53* and *RB1* might have a potential driver function in MPTs [11]. It has also been suggested that *TERT* promoter mutations, either alone [14] or in combination with *MED12* mutations [15], play an important role in the etiology and progression of PTs. Moreover, several less-characterized genetic alterations (e.g. in *ATRX* [16, 17], *BCORL1* [9], and *ZNF217* [11]) whose role in the development and progression of PTs is unknown were identified. As alterations in those genes were rarely reported they are probably not one of the key drivers in the development of PTs. Aside from purely research-driven motivations, a refinement of the genomic profile and subsequent identification of drug targets could create an opportunity for personalized therapy [8].

However, a major challenge in applying next-generation sequencing (NGS) for clinical diagnostics and therapeutic decision-making is the interpretation of identified genetic alterations, in particular variants of unknown significance (VUS) [20, 21]. Several web-based databases (e.g. Catalogue of Somatic Mutations in Cancer, COSMIC [22]), computational algorithms for predicting the impact of mutations on amino acid sequence and protein function (e.g. MutationTaster2 [23]) and knowledge bases incorporating clinical and experimental evidence (e.g. ClinVar [24]) are available free of charge and could aid in the analysis of genetic variants.

Here we present molecular profiling of two patients with MPTs, one with distant and the other with locally recurrent disease. NGS, followed by functional analysis [23, 25] of indicated aberrations, was performed for primary and matched progressed tumor specimens.

## Materials and methods

### Patients

**Fig. 1:**
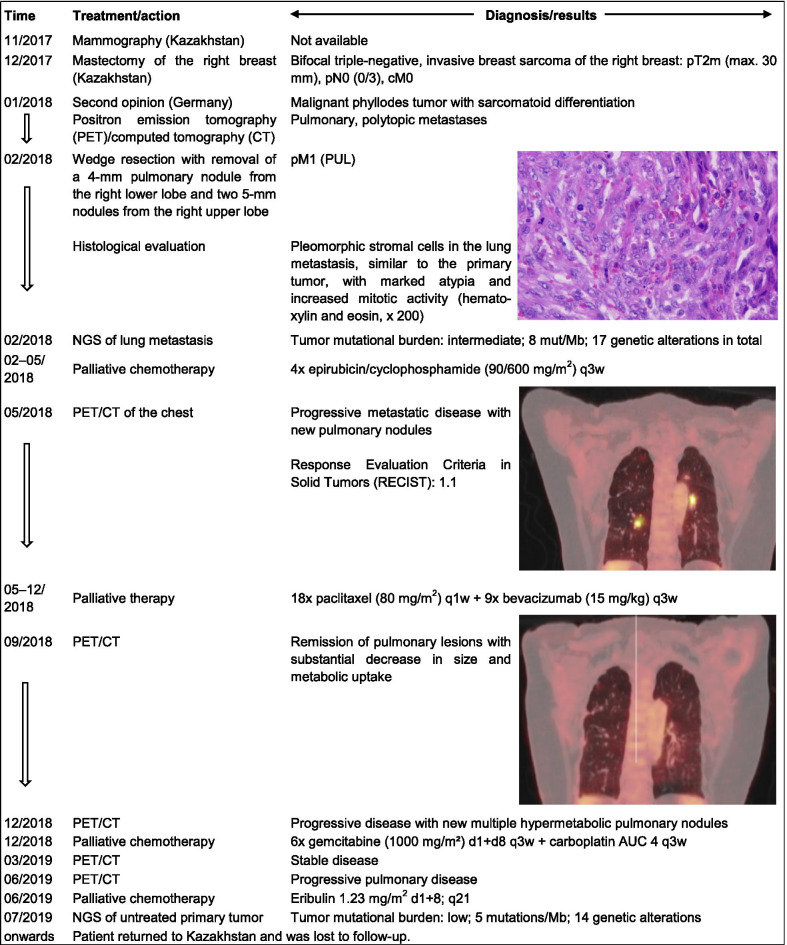
Medical history of a 55-year woman with a metastatic malignant phyllodes tumor (MPT). The patient from Kazakhstan was initially diagnosed with an invasive sarcoma, which was later identified as a MPT following review in the Breast Unit of the Kliniken Essen-Mitte (KEM). In 2009, she had undergone adnexectomy due to the presence of an ovarian cyst. During her examination at the KEM, several pulmonary nodules were detected. Excision and histological characterization of the nodules revealed distant metastases of the MPT. The patient received several lines of chemotherapy treatment and the angiogenesis inhibitor bevacizumab. After her last appointment at the KEM in 06/2019, the patient was lost to follow-up. Next-generation sequencing (NGS) of the metastatic and primary tumor samples was performed

Patient 1, a 55-year-old postmenopausal woman from Kazakhstan, presented herself to our institution one month after she had undergone mastectomy of the right breast and axillary lymph node dissection (Fig. 1) in Kazakhstan where the tumor was initially diagnosed as a triple-negative breast sarcoma (pT2m, pN0 [0/3], cM0) without lymphovascular invasion. She was in good general condition (Karnofsky Performance Status: 100%) and had no family history of PT, although her mother had died of pancreatic cancer at the age of 78. A histological review of slides from the primary tumor (Ki67 index of 70%) indicated a MPT with pleomorphic stromal cells, showing brisk mitotic activity and invasive margins. In the submitted tissue blocks, there was no epithelial or heterologous (e.g. liposarcoma or chondrosarcoma) component. Diagnosis of MPT was made after exclusion of other spindle cell lesions, especially spindle cell carcinoma. Positron emission tomography (PET)/computed tomography (CT) revealed several pulmonary nodules, located in the right upper and lower lobe, which were subsequently excised by a video-assisted wedge resection. Postoperative classification was pM1 (PUL) and was followed by palliative chemotherapy with epirubicin and cyclophosphamide. Three months later, a second PET/CT indicated progressive pulmonary metastatic disease in the right and left lobe. Thereafter, treatment was changed to paclitaxel with bevacizumab. Four months later, the patient presented with complete clinical remission of metastatic disease; however, after a further 3 months, CT of the chest again indicated progressive disease (two pulmonary nodules in the right upper lobe, one nodule in the right lower lobe, and one nodule in the left lower lobe), and the patient began gemcitabine and carboplatin treatment. Under this regimen, she had stable disease for 6 months but due to the progression of the metastatic disease, treatment was changed to eribulin. Thereafter, the patient returned to Kazakhstan and was lost to follow-up.

**Fig. 2:**
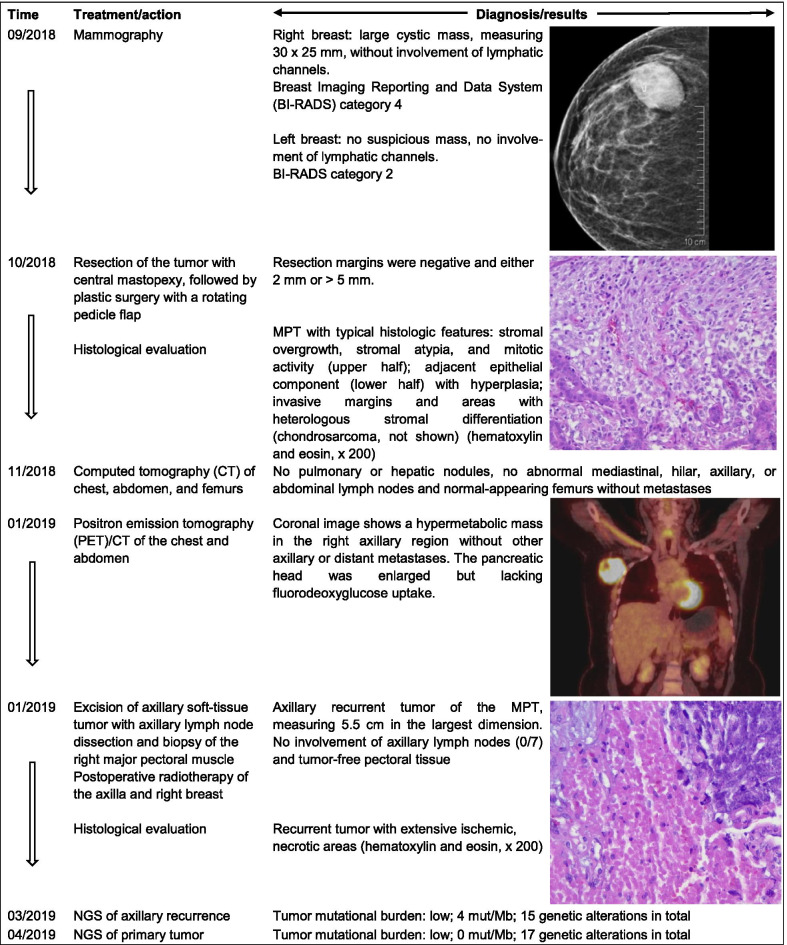
Medical history of a 29-year woman with a recurrent malignant phyllodes tumor (MPT) of the breast (Patient 2). The young woman presented with a lesion in the right breast on mammogram images. Following excision of the lesion, reconstructive surgery was performed. The mass was identified as a MPT without regional or distant spread. Three months later, PET/CT scans demonstrated a large mass in the axillary region. After surgical excision of the lesion and axillary lymph nodes, a node-negative recurrent tumor of the MPT was diagnosed. Adjuvant radiotherapy of the right breast and axilla was administered. Next-generation sequencing (NGS) was performed for both the primary and recurrent MPT. Follow-up examinations showed no local or distant recurrence to date (April 2021)

Patient 2, a 29-year-old premenopausal woman, was in good general condition and had no significant medical history, although an aunt had died of metastatic breast cancer at the age of 45. A cystic mass of 30 × 25 mm was detected on mammogram images (Fig. 2). Thereafter, lumpectomy of the right breast with wide margins was performed, followed by reconstructive surgery with defect coverage using medial and caudal rotations flaps. Histological analysis of the surgical specimen indicated a regressive, cystic, partly necrotic MPT, measuring up to 38 mm in its greatest dimension (pT2). Seven axillary lymph nodes were pathologically negative. There were no distant metastases (cM0), nor was there lymphovascular invasion (L0, V0). Due to insufficient safety margins of less than 10 mm, re-excision surgery had to be performed. Repeated CT scans demonstrated that the chest, abdomen, axilla, and femurs were disease-free. Three months after the primary surgery, PET/CT indicated a large, hypermetabolic mass in the axillary region. Subsequent excision and histological characterization of the lesion (5.5 cm) identified recurrence of the MPT. The patient received postoperative radiotherapy of the right breast and axilla. Regular follow-up exams with ultrasound, mammography, and magnetic resonance imaging did not indicate progression of the disease.

### Ethics statement

According to §15 of the Nordrhein-Westfalen (Germany) Medical Association professional code of conduct, retrospective studies do not require ethics committee approval. Patients provided written informed consent.

### Genomic profiling

Formalin-fixed, paraffin-embedded specimens of the primary tumor (P1) and pulmonary metastases (M1) of Patient 1, and the primary (P2), and recurrent tumor (R2) of Patient 2 were analyzed in a commercial molecular pathology laboratory (Molekularpathologie Südbayern, Penzberg, Germany). Extracted DNA was subjected to NGS utilizing the hybrid capture-based FoundationOne® (sample M1) or FoundationOne® CDx (samples P1, P2, R2) assay (Foundation Medicine Inc., Cambridge, MA, US) as previously described [26]. Focused sequencing with the FoundationOne® assay was conducted for exons of 315 genes and introns of 28 genes, and with the FoundationOne® CDx assay for exons of 324 genes and introns of 36 genes, frequently associated with various neoplasms. The indicated genomic regions were analyzed for base substitutions, insertions and deletions; copy number alterations; rearrangements, translocations; microsatellite instability; and tumor mutational burden (TMB). The routine result report contained a listing of identified gene alterations. Upon request, we received the coding DNA reference sequence, the transcript number, and chromosomal position, as these details were needed for the analysis of sequence variants with in silico prediction tools.

### In silico prediction tools

FATHMM-XF [25] and MutationTaster2 [23] were applied in order to predict the functional effects of identified genetic alterations. FATHMM-XF can be used for functional analysis of non-synonymous single-nucleotide variants (SNVs) and MutationTaster2 can be applied to SNVs as well as insertions and deletions. Predictions with FATHMM-XF are expressed as p-values (range, 0–1) with values close to 0 or 1 yielding predictions with the highest accuracy; values > 0.5 indicate a deleterious SNV and those < 0.5 a neutral or benign SNV. MutationTaster2 predicts pathogenicity of genetic variants as one of four possible types as described in the following types: disease-causing, probably deleterious; disease-causing automatic, known to be deleterious; polymorphism, probably harmless; polymorphism automatic, known to be harmless. To evaluate mutations, chromosomal location (FATHMM-XF) or the position of altered bases in the gene and Ensembl transcript ID (MutationTaster2) were investigated. The Molekularpathologie Südbayern provided relevant data that are not part of a regular FoundationOne® or FoundationOne® CDx report.

## Results

### NGS of paired tumor samples

Alterations in oncogenes or TSGs are depicted in Table 1; alteration in genes, which have not yet been clearly identified as TSGs or oncogenes, are listed in Table 2, with additional details provided in Additional file 1: Table S1. VUS were assigned according to the FoundationOne® (CDx) report. The four investigated MPT samples were microsatellite stable. Samples from Patient 1 were compared with each other; similarly, samples from Patient 2 were compared with each other.

**Table 1:** Alterations in oncogenes or tumor suppressor genes (TSGs) identified in the primary tumor (P1) and lung metastasis (M1) of Patient 1 or the primary tumor (P2) and recurrence (R2) of Patient 2. Variants of unknown significance (VUS) were assigned according to the FoundationOne® (CDx) report

Gene	Oncogene	TSG	Type of alteration			Patient		Prediction of functional consequences		Occurrence of genomic alterations previously reported in phyllodes tumors		
			General classification	Details	VUS	1	2	FATHMM-XF	Mutation Taster2	Specific alteration	Other alterations	Type of tissue
*APC*		+	non-syn. SNV	p.R230H	+	P1, M1		0.98, pathogenic	Disease causing	NR	NR	
*ARID1A*		+	non-fs deletion	p.A345_A349del		M1		–	Polymorphism^a^	NR	One case, non-syn. SNV [18]	P benign
			non-fs insertion	p.A247_G248insA	+		P2, R2	–	Polymorphism	NR		
*BCOR*		+	splicing, stop gain	p.R1031fs*23			P2	–	Disease causing	NR	Few cases [13, 38]	P borderline, malignant
*BRIP1*		+	non-syn. SNV	p.M1V		P1, M1		0.02, benign	Polymorphism	NR	NR	
*CARD11*	+		non-syn. SNV	p.S925C	+		P2, R2	NA	Polymorphism	NR	Few cases: amplification [14, 16]	P malignant
*CASP8*		+	non-syn. SNV	p.F18L	+	P1		NA	Polymorphism	NR	NR	
*CDK4*	+		non-syn. SNV	p.T102K	+	P1, M1		NA	Disease causing	NR	NR	
*EGFR*	+		non-syn. SNV	p.Q432K			P2	0.71, pathogenic	Disease causing	NR	Several non-syn. SNVs [12, 13, 16, 18]	P malignant
			amplification	copy no. = 11			R2	–	–	Several cases [9, 11–14, 18]		P, M malignant
*GRIN2A*	+	+	non-syn. SNV	p.T141K	+	M1		NA	Polymorphism	NR	NR	
*IGF1R*	+		amplification	copy no. = 6			P2	–	–	Two cases [11]		P malignant
				copy no. = 19			R2	–	–			
*KIT*	+		amplification	copy no. = 21			P2	–	–	One case [13]	NR	P malignant
				copy no. = 27			R2	–	–			
*MED12*	+		non-syn. SNV	p.G44V		P1, M1		0.95, pathogenic	Disease causing	Hotspot [9, 11, 13, 14, 18]	High frequency [9, 11, 13, 14, 16, 18]	P, R, M all grades
*KMT2D* *(MLL2)*		+	fs deletion, stop gain	p.E2603fs*88		P1, M1		–	Disease causing	NR	Several cases [9, 13, 16, 38]	P, M all grades
			stop gain	p.Q3293*				NA	Disease causing	NR	Stop gain [13, 14, 16, 18]	
*MYC*	+		amplification	copy no. = 33			P2	–	–	Several cases [10, 11, 13, 18]	NR	P malignant
				copy no. = 44			R2	–	–			
*PAX5*	+	+	non-syn. SNV	p.S213L	+		P2, R2	0.89, pathogenic	Disease causing	NR	One case [38]	P, benign
*PDGFRA*	+		amplification	copy no. = 24			P2	–	–	few cases [11, 13]	NR	R malignant
			amplification	copy no. = 35			R2	–	–			
*RB1*		+	fs deletion, stop gain	I124fs*6			P2	–	Disease causing	NR	Several cases [9–14, 16, 18]	P, M borderline malignant
			stop gain	p. Y321*			P2	NA	Disease causing			
*TERT*	+	+	promoter	c.-124C > T		P1, M1		NA	NA	Hotspot		
[9, 13–15, 18]	high frequency [9, 14–16, 18]	P, R all grades										
*TP53*		+	non-syn. SNV	p.R249S		P1, M1		NA	Disease causing	NR, c.747G > C	High frequency [9, 11–14, 16, 18]	P, R, M mostly malignant
			non-syn. SNV	G262V			P2, R2	1.00, pathogenic	Disease causing	NR		
			non-syn. mutation	p.Q_S6 > HP			R2	–	Polymorphism	NR		
			deletion	exons 2–9			P2, R2	–	–	NR	Deletion of gene [10]	P, distant malignant

^a^polymorphism is probably harmless, according to the definition of MutationTaster2[23]

fs, frameshift; M, distant metastases; NA, not available; NR, not reported; P, primary tumor; R, recurrence; non-syn. SNV, non-synonymous single-nucleotide variant; VUS, variants of unknown significance; *, stop codon

**Table 2:** Variants of unknown significance identified in the primary tumor (P1) and lung metastasis (M1) of Patient 1 as well as the primary (P2) and recurrent tumor (R2) of Patient 2

Gene	Type of alteration		Patient		Prediction of functional consequences		Occurrence of genomic alterations previously reported in PTs		
	General classification	Details	1	2	FATHMM-XF	MutationTaster2	Specific alteration	Other alterations	Type of tissue
*EPHA3*	non-syn. SNV	p.K713T	P1, M1		0.30, benign	Disease causing	NR	NR	
*EPHA7*	non-syn. SNV	p.T118A	M1		0.13, benign^1^	Disease causing	NR	NR	
*EPHB1*	non-syn. SNV	p.R637H	P1, M1		0.06, benign^1^	Disease causing	NR	NR	
*GRM3*	non-syn. SNV	p. N516S		P2, R2	NA	Disease causing	NR	NR	
*MAF*	non-syn. SNV	p.L138M	P1		0.07, benign	Polymorphism	NR	NR	
*MST1R*	non-syn. SNV	p.R470H		P2, R2	0.06, benign^1^	Disease causing	NR	NR	
*NTRK3*	non-syn. SNV	p.S564C	P1, M1		0.02, benign high confidence	Polymorphism	NR	NR	
*PRKN* *(PARK2)*	non-syn. SNV	p.R442G		P2, R2	0.03, benign	Polymorphism	NR	NR	
*PIK3C2G*	non-syn. SNV	p.H1274D		P2, R2	NA	Disease causing	NR	One case, non-syn. SNV [29]	metastatic malignant
*PLCG2*	non-syn. SNV	p.T961M	M1		NA	Disease causing	NR	NR	
*SPTA1*	non-syn. SNV	p.R885H	M1		NA	Polymorphism	NR	NR	
*ZNF703*	fs deletion, stop gain mutation	p.G22fs*50	P1, M1		–	Disease causing	NR	Three cases [18]	P, R: benign, malignant

fs, frameshift; non-syn. SNV, non-synonymous single-nucleotide variant; NA, not available; NR, not reported; P, primary tumor; PT, phyllodes tumor; R, recurrence; M, distant metastases; *, stop codon

^1^According to the COSMIC database (cancer.sanger.ac.uk) [22], the FATHMM-MKL prediction was pathogenic for *EPHA7* (score: 0.94), *EPHB1* (score: 0.99), and *MST1R* (score: 0.95)

**Fig. 3:**
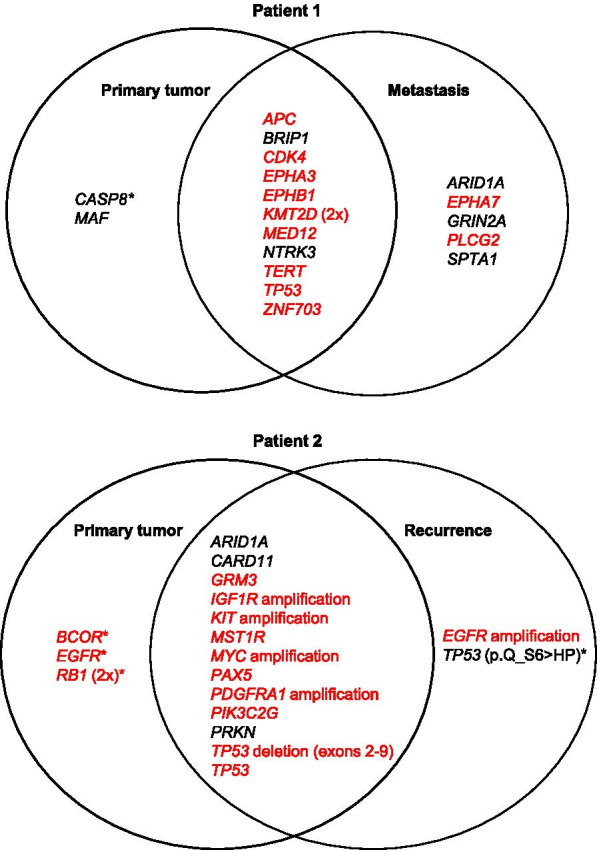
Genes which were altered in the primary or progressed samples or in both are depicted in this schematic diagram. Genetic alterations indicated in red were predicted to be probably deleterious by MutationTaster2. Alterations annotated with a * had mutant allele frequencies < 10%

The untreated primary tumor (P1) and lung metastases (M1) of Patient 1 had a TMB of 5 mut/Mb and 8 mut/Mb, respectively. Fourteen (P1) and 17 (M1) genetic aberrations were identified, of which 12 were present in both lesions (Tables 1 and 2, Fig. 3). P1 and M1 had identical alterations in several TSGs (*APC*, *BRIP1*, *KMT2D*, and *TP53*), oncogenes (*CDK4* and *MED12*), and genes with dual roles in activating or suppressing carcinogenesis such as *GRIN2A* and *TERT* (Table 1). SNVs predicted by MutationTaster2 to be probably disease-causing were identified in *APC*, *CDK4*, *MED12*, *KMT2D*, *TP53*, *PLCG2*, *ZNF703*, and receptor tyrosine kinases (RTKs) *EPHA3* (P1/M1), *EPHB1* (P1/M1), and *EPHA7* (M1). Functional Analysis through Hidden Markov Models (FATHMM)-XF predicted four of these to be benign. *ARID1A*, *EPHA7*, *GRIN2A*, *PLCG2*, and *SPTA1* were altered exclusively in M1, and *MAF* and *CASP8* in P1 (Fig. 3). Both the primary tumor and the pulmonary metastasis harbored a missense mutation in the start codon (p.M1V) of *BRIP1*. Whereas in February 2018 no therapeutic option was indicated, the NGS report from July 2019 recommended off-label treatment with olaparib, which had by then been approved for metastatic breast cancer. At around this time the patient returned to Kazakhstan and was lost to follow-up. It is therefore not clear if she received NGS-based therapy.

In Patient 2, TMB was 0 mut/Mb in the untreated primary tumor (P2) and 4 mut/Mb in the paired recurrent tumor (R2). Seventeen (P2) and 15 (R2) sequence variants were identified, of which 13 were present in both the primary and progressed tumor sample (Tables 1 and 2, Fig. 3). In both P2 and R2, amplification of the RTKs *IGFR1*, *KIT*, and *PDGFRA* as well as the transcription factor *MYC* occurred (Table 1). Moreover, inactivating alterations, such as the deletion of exons 2–9 of *TP53* and nonsense mutations in *RB1*, as well as a SNV in the transcription factor *PAX5* were found in both specimens. Interestingly, amplification of *EGFR* was only identified in the recurrent but not in the primary tumor, suggesting that this might be a crucial event in the evolution of this tumor. Besides alterations in these cancer hallmark genes, SNVs were identified in the less characterized genes *GRM3*, *MST1R*, *PRKN*, and *PIK3C2G* (Table 2). Mutations in genes confined to P2 (*BCOR* and *RB1*) were predicted to be probably disease-causing but only present in low frequencies (Fig. 3, Additional file 1: Table S1).

Four druggable targets (amplification of *EGFR*, *KIT*, and *PDGFRA;* and the missense mutation in *EGFR*) were identified, leading to proposed off-label therapy with the EGFR antibodies cetuximab and panitumumab, several tyrosine kinase inhibitors such imatinib, nilotinib, sunitinib, and afatinib as well as the multi-kinase inhibitor sorafenib. However, due to R0 resection with wide resection margins in Patient 2 and no sign of progressive disease up until April 2021, these recommendations have not yet been adopted.

To the best of our knowledge (Tables 1 and 2), sequence variants in 15 genes found in the two patients described here *(APC, BRIP1*, *CASP8*, *CDK4*, *GRIN2A*, *EPHA3*, *EPHA7*, *EPHB1*, *GRM3*, *MAF*, *MST1R*, *NTRK3, PRKN, PLCG2,* and *SPTA1)* have not previously been associated with PTs. Five of these were in either oncogenes or TSGs. The judgement as to whether genetic alterations were previously reported in PTs was based on the cited references and 561 phyllodes tumors listed in the COSMIC database as of 04/2021 (cancer.sanger.ac.uk) [22].

### Prediction of functional consequences

In 12 out of 25 instances, analysis of a mutation based on the chromosomal location relative to the Genome Reference Consortium Human (GRCh) 38/hg38, as indicated in the raw data of the NGS report (Additional file 1: Table S1), could not be performed by FATHMM-XF as an unexpected reference base was found and therefore results were not available (Tables 1 and 2). MutationTaster2 could make predictions for all mutations except the one in the *TERT* promoter region. Eleven of a total of 38 genetic alterations were found to be probably benign and harmless by MutationTaster2 (e.g. *BRIP1*, *CARD11*, *CASP8*, *NTRK3*, *PRKN*, and *SPAT1)*, including both non-frameshift alterations in *ARID1A*. As MutationTaster2 was considered to predict benignity with high reliability [27], we regarded these predictions as valid. For four of these 11 cases, a FATHMM-XF prediction was available and benign as well. One of the concordant benign predictions with high confidence was for p.M1V in *BRIP1* as detected in Patient 1. The next start codon is at codon 4, and the delayed start of translation possibly results in a shortened protein containing 1247 instead of 1250 amino acids.

The FATHMM-XF score predicted eight SNVs to be benign, whereas Mutationtaster2 classified four of these eight as probably deleterious. Of note, FATHMM-MKL predictions for three of these discordant predictions were available in the COSMIC database and all were pathogenic: *EPHA7* (score: 0.94), *EPHB1* (score: 0.99), and *MSTR1* (score: 0.95) (Table 2).

## Discussion

The application of modern sequencing technologies for the assessment of genomic alterations and actionable targets is especially valuable for rare cancers such as PT, for which the genetic drivers are poorly understood. The present report describes the results of NGS and prediction of functional consequences of identified genetic alterations for paired samples (P1, M1; P2, R2) of two patients with MPTs.

Except for alterations in *ARID1A* and *TP53,* which were present in all samples, the genomic aberrations of the MPTs from Patients 1 and 2 were quite different from each other. The DNA-binding protein *ARID1A* is involved in the regulation of chromatin architecture [28]. It was predicted that the *ARID1A* non-frameshift (non-fs) deletion in M1 and the non-fs insertion in P2 and R2 probably lacked functional consequences. Three different genomic alterations in *TP53* were found in tumor samples from Patient 2. One of these was a deletion of exons 2–9, very likely resulting in a non-functional protein or no TP53 protein at all. *TP53* is a classic TSG involved in many cancer types [29] including PT [10]. It has been suggested that *TP53* alterations play a role in PT progressing from a benign to a malignant histological subtype [12]. *TP53* mutations have been detected mainly in malignant and, to a lesser degree, in borderline PTs [9–13].

Among other alterations, P1 and M1 harbored PT hotspot mutations in *MED12* [11, 13, 18] and the *TERT* promoter region [14, 15] as well as SNVs in *APC* and *CDK4*. The latter are commonly reported in other advanced cancer entities [29] but, to our knowledge, not yet in PTs. It has been pointed out that uterine adenosarcomas and PTs of the breast are both fibroepithelial lesions harboring mutations in members of the Wnt/ß-catenin signaling pathway [17]. The same was true for M1, which had genomic alterations in pathway members *APC*, *TERT*, and *MED12*. MutationTaster2 indicated that the missense mutations detected in *EPHA3*, *EPHA7*, and *EPHB1* were probably disease-causing. The identical SNV (p.R637H, c.1910G > A) in *EPHB1* was previously reported in a patient with breast cancer [30]. Likewise, the identical SNV (p.T118A, c. 352A > G) in *EPHA7* was reported in a colorectal cancer cell line [31]. These Eph receptors belong to a family of 14 RTKs [32], and several of these have been associated with cancer and cancer progression [33].

Paired tumor samples from Patient 2 exhibited a very aggressive genomic pattern as four oncogenes (*EGFR*, *IGF1R*, *MYC*, *KIT*) were amplified and two major TSGs (*TP53*, *RB1*) were deleted or inactivated, a combination likely to promote genomic instability. In this regard, it is not surprising that a recurrent tumor of 5 cm evolved within 3 months after the primary diagnosis and 2 months after a CT documented no suspicious findings in the axillary region. In other investigations, amplifications of *EGFR* [9, 14, 18], *IGF1R* [11], *KIT* [13], *MYC* [10, 11, 13, 18], and *PDGFRA* [11, 12] were confined to MPTs rather than benign or borderline PTs. In fact, it was demonstrated that gene copy alterations were generally associated with higher histological grade [17], suggesting a critical role in the progression of MTPs. Interestingly, four genes encoding RTKs were amplified in Patient 2. A potential actionable target for MPTs is EGFR, which can be blocked by lapatinib. This may offer a potential later therapeutic option for Patient 2, who until now did not show any sign of recurrent disease. In a previous investigation of MPTs, *EGFR* amplification occurred in 8/24 (33%) of cases, while nearly all of them exhibited EGFR protein overexpression [12], suggesting that anti-EGFR therapy could become one of the cornerstones for treatment of MPTs with relevant alterations.

Several of the 12 VUS in genes not classified as TGSs or oncogenes (Table 2) were classified as probably harmless by functional annotation scores. Aberrations for *ZNF703* and *GRM3* were predicted to be probably disease-causing. GRM3 is a G-protein-coupled receptor located upstream of PI3K and the Ras/Raf/MEK/ERK pathway [34]. Of note, none of the 12 VUS reported here has been described in PTs so far, except for *PIK3C2G* and *ZNF703* [18], which belong to the zinc finger protein family of transcription factors. In vitro experiments have shown that *ZNF703* is a negative regulator of Wnt/ß-catenin signaling [35], suggesting that this pathway might play an important role in the carcinogenesis of MPTs.

In a huge NGS project including more than 10,000 patients with metastatic cancer across 62 principal solid tumor entities, the most commonly mutated genes were, in decreasing order of alteration frequency, *TP53*, *KRAS*, *TERT*, *PIK3CA*, *APC*, *ARID1A, PTEN, EGFR, KMT2D,* and *RB1* [29]. Whereas seven of these genes (*TP53, TERT, APC, ARID1A, EGFR, KMT2D,* and *RB1)* were also altered in the two MPTs described here, *PTEN*, *KRAS* and *PIK3CA* had wild-type status. However, *PIK3CA* was reported to be mutated in a mixed cohort of patients with primary and metastatic MPT cases [9, 12], and *PIK3C2G* (mutated in P2, R2) belongs, like *PIK3CA,* to the PI3K family. PTEN is an upstream regulator of the PI3K/AKT/mTOR signaling pathway [36], and was previously shown to be mutated in MPTs [13]. In addition, ligand-independent upregulation of RTKs (e.g. *EGFR*, *PDGFR, KIT*) due to gain-of-function mutations (in P2 and R2) can result in constitutive downstream activation of the Ras/Raf/MEK/ERK pathway, ultimately leading to proliferation and apoptosis resistance [37]. Therefore, despite the limited knowledge regarding underlying mutational events in MPTs, it seems that most of the major genomic alterations frequently occurring in other advanced cancer types are involved in this process as well. These findings should be confirmed in a larger cohort.

A limitation of our evaluation is that the application of in silico prediction tools is not without controversy; a main point of criticism is that predictions might be false positives in some instances even when several prediction tools were applied [27]. In one study, which tested the performance of four prediction tools, MutationTaster2 predicted no false-negative results and was therefore considered a suitable algorithm to predict benignity [27]. Regarding practicability, MutationTaster2 definitely outperformed FATHMM-XF as it predicted functional consequences in 34 of 35 SNVs, insertions or deletions, whereas the FATHMM-XF score could only be obtained for 13 out of 25 SNVs. These findings are based on raw data we requested for the original FoundationOne® (CDx) reports; we therefore do not have further insight into the functionality of this prediction tool. Despite the drawbacks associated with these algorithms, especially for clinical decision-making, they added further information to NGS results presented here. Prediction of functional consequences was especially helpful for the interpretation of VUS and particularly in genes which have not been associated with MPTs and/or whose role in carcinogenesis in general is not well described yet.

## Conclusions

Analysis of data generated by NGS provided new insights into the molecular pathogenesis of recurrent and metastatic MPTs, identified novel mutations involved in the progression of MPTs, discovered remarkable similarity with the 10 most frequently altered genes in other advanced cancer entities, and suggested potential therapeutic options. Merely listing genomic alterations without functional analysis could be misleading, as several alterations seem to be benign and might have no role in the pathogenesis of PT.

## Supplementary Information

**Additional file 1: Table S1.** Details of genetic alterations identified in the primary tumor (P1) and lung metastasis (M1) of Patient 1 and the primary (P2) and recurrent (R2) tumors of Patient 2, based on the Ch38 (hg38) version of the human genome assembly.

## Data Availability

All data generated or analyzed during this study are included in this published article [and its supplementary information files].
