# Supplementary material for: Two progressed malignant phyllodes tumors of the breast harbor alterations in genes frequently involved in other advanced cancers
Source: Orphanet J Rare Dis. 2021 Aug 16;16:363. doi: 10.1186/s13023-021-01986-z (PMC8365991; doi:10.1186/s13023-021-01986-z)
Supplement: Supplementary file 1 — Additional file 1: Table S1. Details of genetic alterations identified in the primary tumor (P1) and lung metastasis (M1) of Patient 1 and the primary (P2) and recurrent (R2) tumors of Patient 2, based on the Ch38 (hg38) version of the human genome assembly. [file 13023_2021_1986_MOESM1_ESM.docx]

**Table S1** Details of genetic alterations identified in the primary tumor (P) and lung metastasis (M) of Patient 1 and the primary (P) and

recurrent (R) tumor of Patient 2, based on the Ch38 (hg38) version of the human genome assembly.

| **Gene** | **Type of alteration** | | **Case 1** | **Case 2** | **Mutation allele frequency** | **Transcript** | **Chromosomal**  **position** |
| --- | --- | --- | --- | --- | --- | --- | --- |
|  | **Protein effect** | **CoDing Sequence** |  |  |  |  |  |
| *APC* | p.R230H | c.689G>A | P, M |  | 7.3%, 34.0% | NM_000038 | chr5:112128186 |
| *ARID1A* | p.A345_A349del | c.1029_1043delAGC TGCGGCGGCGGC | M |  | 41.7% | NM_006015 | chr1:27023922 |
|  | p.A247_G248insA | c.737_738insGGC |  | P, R | 44.0%, 38.1% | NM_006015 | chr1:27023631 |
| *BCOR* | p.R1031fs*23 | c.3090_3093delAAGA |  | P | 6.8% | NM_017745 | chr23: 39930370 |
| *BRIP1* | p.M1V | c.1A>G | P, M |  | 56.1%, 50.2% | NM_032043 | chr17:59938900 |
| *CARD11* | p.S925C | c.2773A>T |  | P, R | 71.7%, 87.9% | NM_032415 | chr7:2954937 |
| *CASP8* | p.F18L | c.52T>C | P |  | 8.4% | NM_001080125 | chr2:202123006 |
| *CDK4* | p.T102K | c.305C>A | P, M |  | 44.2%, 19.1% | NM_000075 | chr12:58145039 |
| *EGFR* | p.Q432K | c.1294C>A |  | P | 9.6% | NM_005228 | chr7:55225442 |
| *EPHA3* | p.K713T | c.2138A>C | P, M |  | 45.0%, 53.7% | NM_005233 | chr3:89480301 |
| *EPHA7* | p.T118A | c.352A>G | M |  | 63.6% | NM_004440 | chr6:94120699 |
| *EPHB1* | p.R637H | c.1910G>A | P, M |  | 44.6%, 16.3% | NM_004441 | chr3:134911445 |
| *GRIN2A* | p.T141K | c.422C>A | M |  | 44.3% | NM_000833 | chr16:10032401 |
| *GRM3* | p. N516S | c.1547A>G |  | P, R | 45.2%, 40.4% | NM_000840 | chr7:86468377 |
| *KMT2D*  *(MLL2)* | p.E2603fs*88 | c. 7803delA | P, M |  | 44.9%, 18.8% | NM_003482 | chr12:49433749 |
|  | p.Q3293* | c.9877C>T |  |  | 42.4%, 17.1% | NM_003482 | chr12:49431262 |
| *MAF* | p.L138M | c.412C>A | P |  | 45.7% | NM_005360 | chr16:79633388 |
| *MED12* | p.G44V | c.131G>T | P, M |  | 44.6%, 15.1% | NM_005120 | chrX:70339254 |
| *MST1R* | p.R470H | c.1409G>A |  | P, R | 49.5%, 49.6% | NM_002447 | chr3:49936518 |
| *NTRK3* | p.S564C | c.1690A>T | P, M |  | 52.1%, 46.5% | NM_001007156 | chr15:88524487 |
| *PAX5* | p.S213L | c.638C>T |  | P, R | 54.3%, 51.2% | NM_016734 | chr9:36966688 |
| *PRKN*  *(PARK2)* | p.R442G | c.1324A>G |  | P, R | 54.9%, 49.4% | NM_004562 | chr6:161771205 |
| *PIK3C2G* | p.H1274D | c.3820C>G |  | P, R | 70.2%, 84.2% | NM_004570 | chr12:18719923 |
| *PLCG2* | p.T961M | c.2882C>T | M |  | 49.1% | NM_002661 | chr16:81969813 |
| *RB1* | p.I124fs*6 | c. 371_372delTA |  | P | 5.3% | NM_000321 | chr13:48916840 |
|  | p. Y321* | c.963C>A |  | P | 9.8% | NM_000321 | chr13:48941653 |
| *SPTA1* | p.R885H | c.2654G>A | M |  | 43.0% | NM_003126 | chr1:158627418 |
| *TERT* | promoter region | c.-124C>T | P, M |  | 37.7%, 12.1% | NM_198253 | chr5:1295228 |
| *TP53* | p.R249S | c.747G>C | P, M |  | 89.4%, 43.2% | NM_000546 | chr17:7577534 |
|  | p.G262V | c.785G>T |  | P, R | 53.4%, 61.0% | NM_000546 | chr17:7577153 |
|  | p.Q_S6>HP | c.15_16GT>TC |  | R | 1.6% | NM_000546 | chr17:7579897 |
| *ZNF703* | p.G22fs*50 | c.64_92del29 | P, M |  | 28.8%, 15.2% | NM_025069 | chr8:37553560 |
